# Significant shortening of leukocyte telomeres one year after childbirth in women with type 1 diabetes mellitus: a prospective cohort study

**DOI:** 10.3389/fcdhc.2026.1776531

**Published:** 2026-03-12

**Authors:** Mislav Herman, Marina Ivanisevic, Josip Djelmis

**Affiliations:** Referral Centre Ministry of Health, Department of Obstetrics and Gynecology, University Hospital Centre Zagreb, School of Medicine University of Zagreb, Zagreb, Croatia

**Keywords:** biological aging, body mass index, HbA1c, leukocyte telomere length, postpartum, pregnancy, telomere length, type 1 diabetes mellitus

## Abstract

**Background:**

Telomere shortening is influenced by biological and environmental factors, which are especially pronounced during and after pregnancy in women with type 1 diabetes mellitus (T1DM). This study investigates the dynamics of maternal leukocyte telomere length (LTL) during pregnancy and the postpartum period in women with T1DM.

**Methods:**

A prospective cohort of 74 pregnant women with T1DM was enrolled from February 2019 to January 2021 in Zagreb, Croatia. Leukocyte telomere length (LTL), measured as T/S ratio by quantitative PCR, was assessed at delivery (n=74) and one year postpartum (n=46).

**Result:**

At delivery, median LTL was 2.64 (IQR: 1.87-4.59). One year postpartum, median LTL decreased significantly to 1.87 (IQR: 1.23-2.46), representing a 29% relative reduction (P < 0.001, Wilcoxon signed-rank test, r = 0.72). Neither HbA1c levels nor BMI significantly influenced LTL at delivery. No significant difference in LTL was observed between term and preterm deliveries.

**Conclusions:**

Women with T1DM experience significant telomere shortening within the first year postpartum. While HbA1c and BMI showed no significant effects in this cohort, they remain potential modifiers of telomere dynamics and merit further investigation. These findings highlight the need for targeted postpartum care strategies in this high-risk population.

## 1 Introduction

Telomeres are specialized nucleoprotein structures located at the end of eukaryotic chromosomes, consisting of repetitive TTAGGG sequences and associated proteins that protect chromosomal ends from degradation and aberrant recombination (1, 2). These structures are essential for maintaining genomic stability and cellular function, serving as a molecular clock that reflects cellular replicative history. With each cell division, telomeres progressively shorten due to the end-replication problem, ultimately leading to replicative senescence when critically short lengths are reached (3).

The rate of telomere attrition is influenced by numerous biological and environmental factors, including oxidative stress, chronic inflammation, metabolic disturbances, and lifestyle factors (4, 5). Accelerated telomere shortening has been associated with various chronic diseases and has emerged as a biomarker of biological aging that may provide insights into disease progression and mortality risk (6).

Pregnancy represents a unique physiological state characterized by profound hormonal fluctuations, immunological adaptations, and metabolic changes that place substantial demands on maternal physiology (7). Recent evidence suggests that pregnancy and the postpartum period may accelerate biological aging processes, including telomere shortening, though the mechanisms underlying this phenomenon remain incompletely understood (8, 9).

Type 1 Diabetes Mellitus (T1DM) is a chronic autoimmune disease characterized by the destruction of pancreatic β-cells, resulting in absolute insulin deficiency and consequent hyperglycemia (10). The metabolic dysregulation inherent to T1DM creates a milieu of persistent oxidative stress and chronic low-grade inflammation, both of which are established drivers of telomere shortening (11, 12). A negative correlation has been observed between telomere length and T1DM, with several studies demonstrating shorter telomere length in individuals with T1DM compared to healthy controls (13).

The intersection of pregnancy and T1DM presents a particularly challenging clinical scenario, as the metabolic demands of gestation are superimposed upon the pre-existing metabolic burden of diabetes. However, limited data exist regarding the trajectory of telomere length in women with T1DM during the perinatal period (14).

This prospective cohort study aimed to investigate leukocyte telomere length (LTL) dynamics in mothers with T1DM at the time of delivery and one year postpartum, with particular attention to the influence of glycemic control and maternal body mass index on telomere attrition patterns.

## 2 Materials and methods

### 2.1 Study design and participants

This prospective cohort study was conducted at the Referral Centre Ministry of Health, Department of Obstetrics and Gynecology, Clinical Hospital Centre Zagreb, School of Medicine, University of Zagreb, Croatia. Enrollment occurred from February 1, 2019 to January 31, 2021. The study period partially overlapped with the COVID-19 pandemic; however, only 4 of 74 participants were diagnosed with COVID-19, all presenting with mild symptoms not requiring hospitalization, and none were included in the longitudinal cohort.

Eligible participants were pregnant women with established T1DM who had singleton pregnancies and were recruited before completing 10 gestational weeks. Inclusion criteria required insulin therapy for at least two years prior to conception and HbA1c ≤8% (64 mmol/mol) at pregnancy confirmation. All participants received intensified insulin therapy with fast-acting and long-acting insulin formulations.

The universal cesarean delivery in our cohort reflects the clinical practice at our referral center, where elective cesarean section is the standard of care for women with T1DM to optimize timing of delivery and minimize neonatal complications associated with diabetic pregnancies (macrosomia, shoulder dystocia, and neonatal hypoglycemia).

Exclusion criteria included proliferative retinopathy, nephropathy, chronic hypertension, age under 18 years, and major fetal defects identified on 11-13-week ultrasound examination. All deliveries were performed via cesarean section.

### 2.2 Data collection

Demographic and clinical data collected included maternal age, duration of T1DM, age at disease onset, educational level, pre-pregnancy BMI (calculated from self-reported pre-pregnancy weight and measured height), gestational weight gain, BMI at delivery, and HbA1c levels in the first and third trimesters. Neonatal outcomes included birth weight, length, gestational age at delivery, and Apgar scores at 1 and 5 minutes.

All participants were admitted to the Department of Obstetrics and Gynecology at least once or repeatedly in each trimester. Daily glucose profiles were determined, with plasma glucose monitored nine times daily for 2–3 days. Glucose was measured in capillary plasma at the following time intervals: 7, 10, 13, 16, 19, 22, 1, 4, and 7 hours.

### 2.3 Blood sample collection and DNA isolation

Peripheral blood samples (3 mL) were collected from 74 mothers at the time of cesarean delivery and from 46 mothers at cubital venipuncture one year postpartum. Samples were stored in EDTA tubes at -80 °C until DNA extraction. DNA was isolated from whole blood using a protocol based on protein precipitation at high salt concentrations, followed by purification with the NucleoSpin gDNA Clean-up kit (Machery-Nagel, Düren, Germany) according to manufacturer instructions. DNA quality and concentration were assessed using a NanoDrop ND-2000 spectrophotometer (NanoDrop Technologies, Wilmington, DE, USA). Each sample was diluted to a working concentration of 20 ng/µL and stored at -20 °C until analysis.

### 2.4 Telomere length measurement

LTL was measured using quantitative polymerase chain reaction (qPCR) based on the method described by Cawthon (15) and modified by Joglekar et al. (16). Human β-globin was used as a single-copy reference gene due to its stability across cell types and low inter-sample variability.

The T/S ratio was calculated using the 2^−ΔΔCt^ method, where:


ΔCtsample = Cttelomere − Ctβ−globin

ΔΔCt = ΔCtsample − ΔCtcalibrator

$$\text{T}/\text{S ratio }=\;2^{-\Delta \Delta \text{Ct}}$$

qPCR reactions were performed in triplicate using 1 µL of isolated DNA (20 ng/µL), Fast SYBR Green master mix (ThermoFisher Scientific, Waltham, MA, USA), and 10 µM forward and reverse primers. Each plate included triplicate no-template controls (NTC), control DNA (Promega, Madison, WI, USA), and experimental samples for both telomere and β-globin primers. Amplification was performed on a CFX96 Touch real-time PCR detection system (Bio-Rad Laboratories, Hercules, CA, USA) with an optimized annealing temperature of 58 °C for both primer pairs.

### 2.5 Laboratory analyses

Glucose quantification was carried out using the hexokinase method on a Cobas C301 analyzer (Roche, Basel, Switzerland). Measurement of HbA1c levels in whole blood was conducted through turbidimetric inhibition immunoassays on a Cobas C501 instrument (Roche, Basel, Switzerland).

### 2.6 Statistical analysis

Sample size was determined based on preliminary data with the objective of achieving minimum statistical power of 80% (α=0.05). For the pilot study, 28 participants were analyzed. Continuous variables were assessed for normality and are presented as median with interquartile range (IQR) for non-normally distributed data. Between-group comparisons were performed using the Mann-Whitney U test, while within-subject comparisons between delivery and one-year postpartum were analyzed using the Wilcoxon signed-rank test for matched pairs. Effect sizes are reported as rank-biserial correlations (r) for non-parametric tests, with r = 0.1, 0.3, and 0.5 corresponding to small, medium, and large effects, respectively. Median differences were estimated using the Hodges-Lehmann estimator with 95% confidence intervals calculated via the exact method. For regression analyses, both unstandardized (β) and standardized coefficients are reported; standardized coefficients represent effect sizes in standard deviation units. Bootstrapped 95% confidence intervals (10,000 resamples) were calculated for percent change estimates. Participants with missing data for specific outcomes were excluded from the relevant analyses. A P-value <0.05 was considered statistically significant. All analyses were conducted using SPSS version 26 (IBM, Armonk, NY, USA) with effect size calculations performed in R version 4.2 (R Foundation for Statistical Computing, Vienna, Austria).

### 2.7 Ethical considerations

The study was approved by the Ethics Committee at the School of Medicine, University of Zagreb (No. 380-59-10106-19-111/26). All participants provided written informed consent for themselves and their neonates. The study funder had no role in study design, data collection, analysis, interpretation, or manuscript preparation.

## 3 Results

### 3.1 Participants flow

**Figure 1:**
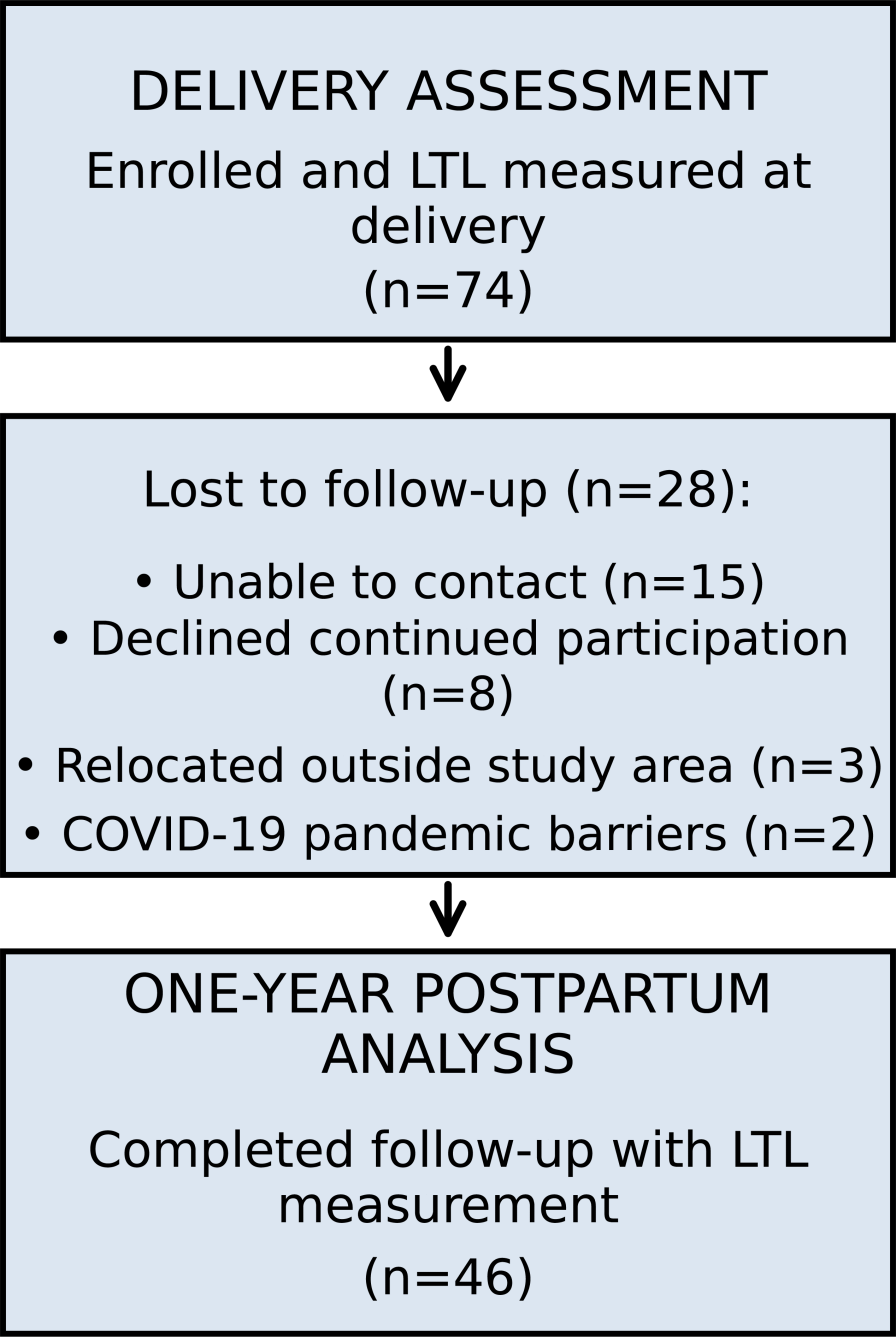
Study flow diagram. Flow diagram of participant enrollment, follow-up, and analysis.

Of the 74 participants with LTL measured at delivery, 46 (62.2%) completed one-year postpartum follow-up. Reasons for loss to follow-up included inability to contact (n=15), declined continued participation (n=8), relocation outside the study area (n=3), and COVID-19 pandemic-related barriers (n=2) (**Figure 1**).

### 3.2 Participant characteristics

Participant characteristics are summarized in **Table 1**. The median maternal age was 31 years (IQR 26-34), ranging from 19 to 40 years. Duration of T1DM ranged from 2 to 37 years (median 15 years, IQR 6-21), with age at disease onset ranging from 2 to 36 years (median 14 years, IQR 9-24).

**Table 1:** Maternal and neonatal demographics, obstetric, and laboratory data.

Variable	N (%)	Min	Max	Median	IQR
Maternal age (years)	74	19	40	31	26-34
Duration of T1DM (years)	74	2	37	15	6-21
Age at T1DM onset (years)	74	2	36	14	9-24
Maternal height (cm)	74	156	183	166	162-170
Pre-pregnancy BMI (kg/m²)	74	15.1	39.8	22.9	20.4-26.0
Gestational weight gain (kg)	74	0	22	13	10-16
BMI at delivery (kg/m²)	74	17.9	43.0	27.5	25-31.1
HbA1c 1st trimester (%)	74	5.1	8.0	6.6	6.0-7.3
HbA1c 3rd trimester (%)	74	4.2	8.6	6.0	5.5-6.5
LTL at delivery (T/S)	74	0.71	26.0	2.64	1.87-4.59
LTL one year postpartum (T/S)	46	0.62	8.0	1.87	1.23-2.46
Gestational age (weeks)	74	32	39	38	38-38
Term birth	66 (89.2)	–	–	–	–
Preterm birth	8 (10.8)	–	–	–	–
Neonatal weight (g)	74	1490	4840	3605	3200-4040
Neonatal length (cm)	74	40	54	49	48-51
Apgar score at 1 min	74	8	10	10	10-10
Apgar score at 5 min	74	6	10	10	10-10

BMI, body mass index; LTL, leukocyte telomere length; T/S, telomere to single-copy gene ratio.

Educational attainment was high in this cohort, with 45.9% having completed high school or university and 54.1% holding graduate degrees. Pre-pregnancy BMI ranged from 15.1 to 39.8 kg/m² (median 22.9, IQR 20.4-26.0), with a median gestational weight gain of 13 kg (IQR 10-16). At delivery, median BMI was 27.5 kg/m² (IQR 25-31.1). Glycemic control was generally good, with median HbA1c of 6.6% in the first trimester (IQR 6.0-7.3) and 6.0% in the third trimester (IQR 5.5-6.5).

### 3.3 Obstetric and neonatal outcomes

The majority of deliveries were at term (66/74, 89.2%), with 8 preterm births (10.8%). Median gestational age was 38 weeks (IQR 38-38). Al deliveries were by cesarean section. Neonatal birth weight ranged from 1490 to 4840 g (median 3605 g, IQR 3200-4040), and neonatal length ranged from 40 to 54 cm (median 49 cm, IQR 48-51). Neonatal outcomes were generally favorable, with median Apgar scores of 10 at both 1 and 5 minutes.

### 3.4 Telomere length at delivery

#### 3.4.1 Baseline measurements

At delivery, median LTL for the entire cohort was 2.64 T/S ratio (IQR 1.87–4.59), with values ranging from 0.71 to 26.0 T/S ratio. Values below 1.0 (n=2) represent samples with telomere content below the calibrator reference; a T/S ratio of 0.71 corresponds to 71% of the calibrator telomere content.

#### 3.4.2 LTL by maternal BMI

**Figure 2:**
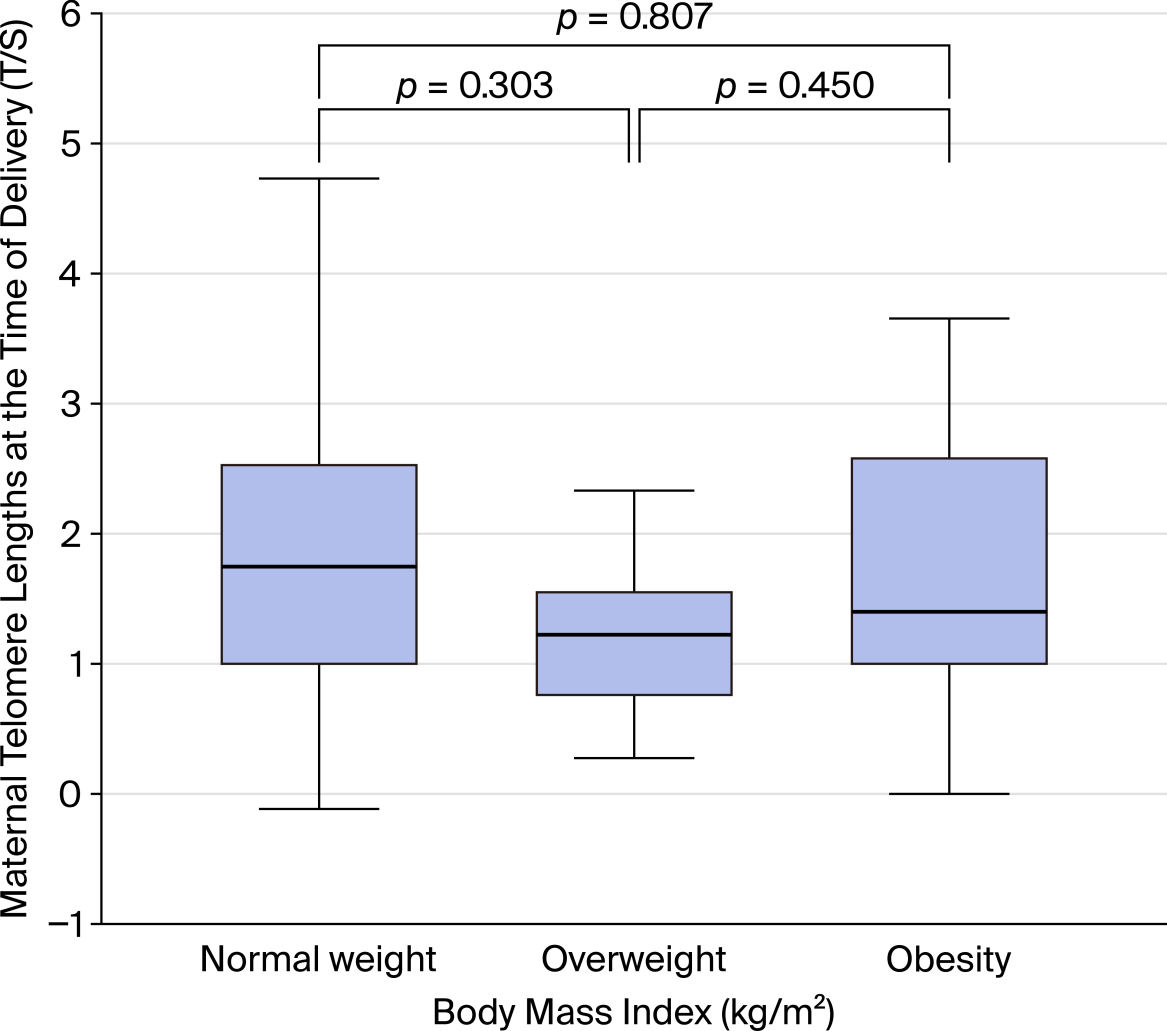
Leukocyte telomere length (LTL) at delivery stratified by maternal body mass index (BMI) category. Box plots show median and interquartile range. Normal weight (BMI <25 kg/m², n=20), overweight (BMI 25-29.9 kg/m², n=30), obese (BMI ≥30 kg/m², n=24). No significant differences were observed between groups.

Normal-weight mothers (n=20) had median LTL of 3.48 T/S ratio (IQR 2.00–7.46), overweight mothers (n=30) had median LTL of 2.64 T/S ratio (IQR 1.74–3.25), and obese mothers (n=24) had median LTL of 2.64 T/S ratio (IQR 2.00–6.06). The median difference between normal weight and overweight/obese mothers was 0.84 T/S ratio (Hodges-Lehmann estimator, 95% CI: 0.87–1.84), with a small-to-medium effect size (rank-biserial r = 0.22, 95% CI: −0.08 to 0.48, P = 0.14; **Figure 2**).

#### 3.4.3 LTL by glycemic control

**Figure 3:**
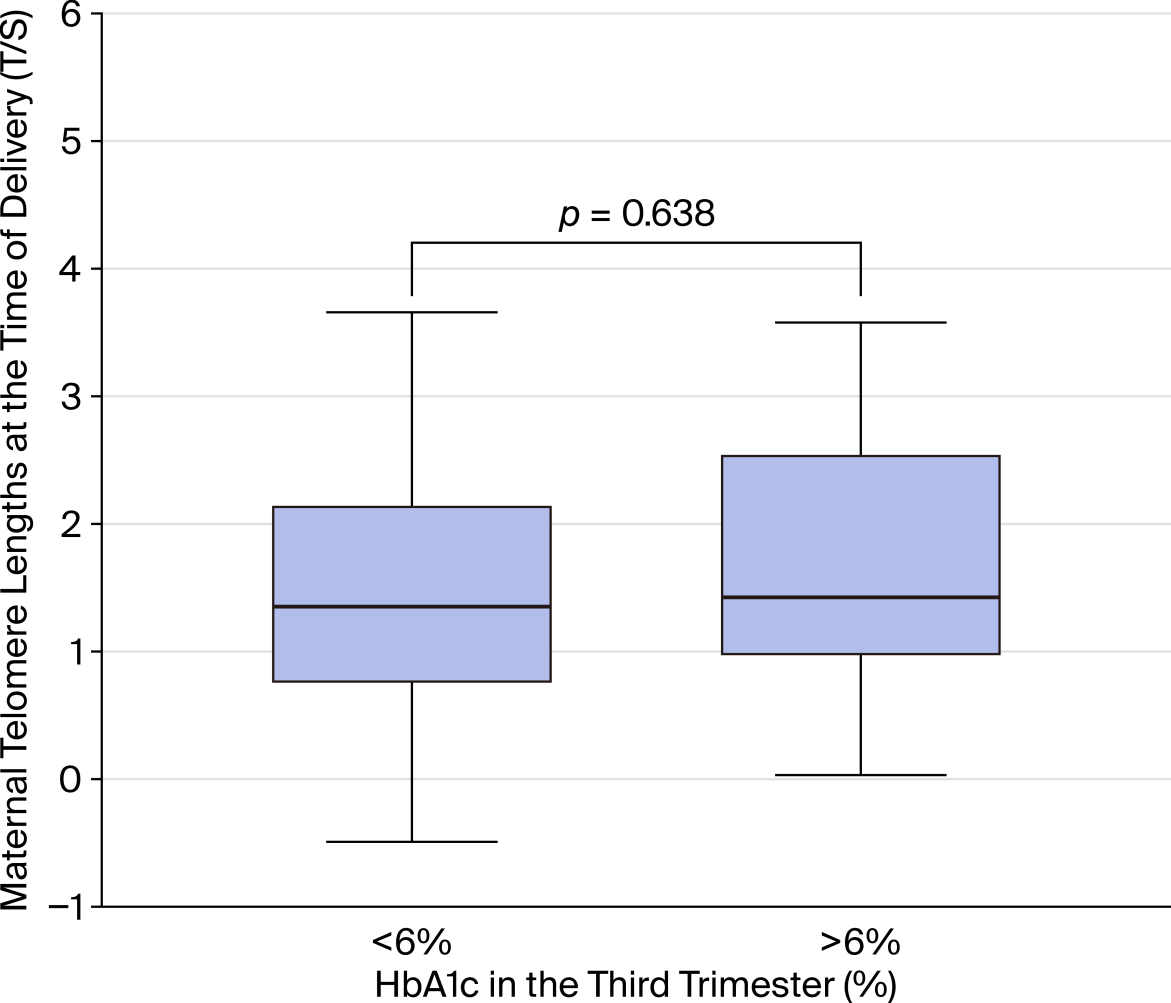
Leukocyte telomere length (LTL) at delivery stratified by third-trimester HbA1c level. Box plots show median and interquartile range. HbA1c <6% (n=41) versus HbA1c ≥6% (n=33). P = 0.638 by Mann-Whitney U test.

Mothers with third-trimester HbA1c <6% (n=41) had median LTL of 2.64 T/S ratio (IQR 1.74–4.00), compared to 2.83 T/S ratio (IQR 2.14–7.46) in those with HbA1c ≥6% (n=33). The median difference was −0.19 T/S ratio (95% CI: −1.50 to 1.30), with a negligible effect size (r = 0.06, 95% CI: −0.22 to 0.34, P = 0.64; **Figure 3**). The confidence interval is consistent with no effect of glycemic control category on LTL at delivery in this well-controlled cohort.

#### 3.4.4 LTL by gestational age at delivery

**Figure 4:**
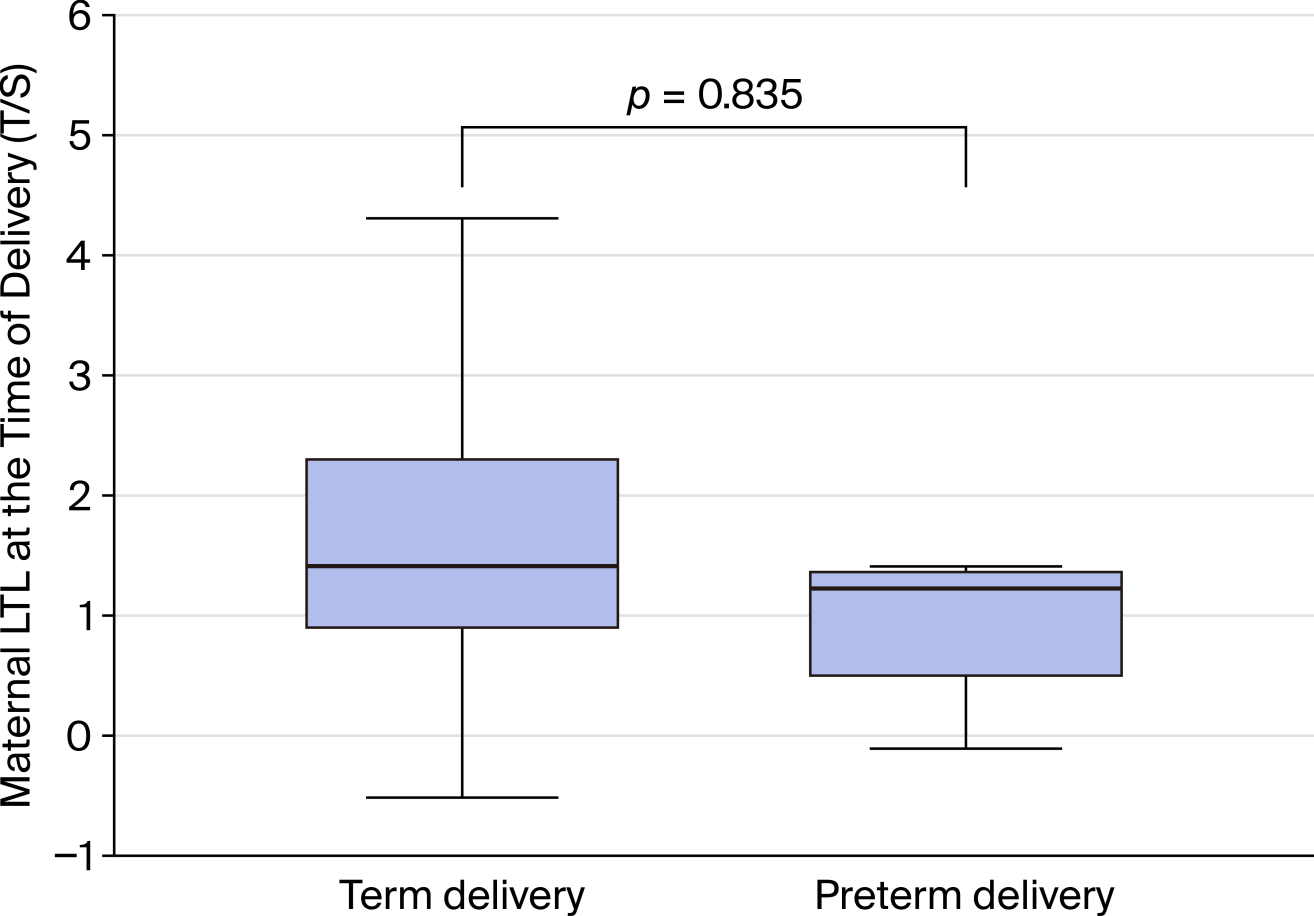
Leukocyte telomere length (LTL) at delivery stratified by gestational age. The study included 66 term births and 8 preterm births. Box plots show median and interquartile range. The median telomere length for mothers who gave birth at term was 1.4, with an interquartile range (IQR) of 0.9 to 2.3. In contrast, the median telomere length for mothers who experienced preterm births was 1.3, with an IQR of 0.5 to 1.4. The difference between these groups was not statistically significant, with a P-value of 0.835.

Mothers delivered at term (n=66) had median LTL of 2.64 T/S ratio (IQR 1.87–4.92) compared to 2.46 T/S ratio (IQR 1.41–2.64) in mothers with preterm delivery (n=8). The median difference was 0.18 T/S ratio (95% CI: −1.57 to 1.80), with a negligible effect size (r = 0.04, 95% CI: −0.45 to 0.53). The small number of preterm deliveries resulted in wide confidence intervals, limiting statistical power (**Figure 4**).

### 3.5 Postpartum telomere shortening

#### 3.5.1 Primary outcome: telomere length change from delivery to one year postpartum

Among the 46 participants with paired measurements, LTL decreased significantly from delivery to one year postpartum (median 2.64 T/S ratio, IQR 1.87–4.59 vs. median 1.87 T/S ratio, IQR 1.23–2.46).

The median paired difference was 0.77 T/S ratio (Hodges-Lehmann estimator, 95% CI: 0.55 to 1.22), representing a 29% relative reduction from delivery values (95% CI: 21% to 38%). The matched-pairs rank-biserial correlation was r = 0.72 (95% CI: 0.54 to 0.84), indicating a large effect size (Wilcoxon signed-rank test, P < 0.001).

Of the 46 participants, 39 (84.8%) showed telomere shortening, 5 (10.9%) showed lengthening, and 2 (4.3%) showed no measurable change.

#### 3.5.2 Predictors of postpartum telomere shortening

To explore potential determinants of postpartum telomere attrition, we performed multivariate linear regression with ΔLTL (delivery minus one-year postpartum) as the dependent variable (**Table 2**). After adjusting for maternal age, T1DM duration, third-trimester HbA1c, gestational weight gain, and pre-pregnancy BMI, none of the examined covariates significantly predicted telomere shortening (all standardized β < 0.20, indicating small effects). The model explained only 8% of the variance (adjusted R² = 0.08), indicating that unmeasured factors contribute substantially to postpartum telomere dynamics in this population.

**Table 2:** Linear regression for LTL change (ΔLTL = LTL delivery – LTL postpartum).

Variable	β coefficient	95% CI	P-value
Maternal age (years)	−0.02	−0.06, 0.02	0.34
T1DM duration (years)	0.01	−0.02, 0.04	0.58
HbA1c 3rd trimester (%)	0.12	−0.18, 0.42	0.41
Gestational weight gain (kg)	−0.03	−0.08, 0.02	0.22
Pre-pregnancy BMI (kg/m²)	0.02	−0.04, 0.08	0.49

Adjusted R² = 0.08; none of the examined covariates significantly predicted the magnitude of telomere shortening

## 4 Discussion

This prospective cohort study demonstrates that women with T1DM experience marked leukocyte telomere shortening within the first year postpartum. These findings extend previous evidence that pregnancy and the postpartum period represent biologically demanding phases that can accelerate biological aging, particularly in women with chronic conditions such as T1DM (17, 18). The significant reduction in LTL observed across the cohort suggests that cumulative exposure to hyperglycemia, systemic inflammation, and oxidative stress—central features of T1DM—exerts long-term biological stress, which becomes particularly evident in the postpartum period (13, 19, 20).

In the absence of a contemporaneous control group, we contextualize our findings against published literature on postpartum telomere and biological aging dynamics. A recent systematic review by Houminer-Klepar et al. (21) examined telomere length changes throughout pregnancy and postpartum across 14 studies. Of the four longitudinal studies measuring telomere length from early pregnancy to early postpartum, three reported no significant change, suggesting that pregnancy itself may not substantially alter maternal telomere length in healthy women over this relatively short interval. However, these studies measured only early postpartum (typically 6–12 weeks), not one year as in our study. The 36% median reduction in T/S ratio observed in our T1DM cohort contrasts sharply with these findings of stability or recovery in healthy populations, suggesting that diabetes-specific factors including chronic oxidative stress, inflammation, glycemic variability, and potentially impaired recovery capacity may accelerate postpartum telomere shortening and prevent the biological rejuvenation observed in healthy women. Direct comparison across studies is limited by differences in measurement techniques, timing of postpartum sampling, and population characteristics; definitive conclusions regarding the excess burden attributable to T1DM require future case-control designs with matched healthy pregnant women using identical methodology.

The postpartum period in women with T1DM presents unique biological stressors that may accelerate telomere attrition through multiple interconnected pathways. First, the rapid withdrawal of placental hormones (estrogen, progesterone, human placental lactogen) removes their established antioxidant and telomere-protective effects, potentially exposing telomeric DNA to increased oxidative damage during a period of heightened metabolic stress. Second, postpartum insulin requirements fluctuate substantially in T1DM, with many women experiencing unpredictable glycemic excursions during the transition from pregnancy-adapted insulin regimens; emerging evidence suggests that glucose variability may be more detrimental to telomere integrity than sustained moderate hyperglycemia alone, as rapid oscillations induce repeated oxidative stress cycles (22). Third, while breastfeeding confers metabolic benefits, the increased energy demands may exacerbate oxidative stress in the context of diabetes, particularly when coupled with the sleep deprivation inherent to newborn care. Fourth, postpartum sleep disruption and psychosocial stress are well-established accelerators of telomere shortening in the general population (18), and may be amplified in mothers managing the dual demands of infant care and chronic disease self-management. Finally, postpartum depression which occurs at elevated rates in women with T1DM compared to the general postpartum population has been independently associated with accelerated telomere attrition and may represent an underrecognized contributor to biological aging in this group.

The clinical significance of accelerated postpartum telomere shortening extends beyond the abstract concept of biological aging to encompass tangible long-term health consequences. In T1DM populations, shorter telomere length independently predicts all-cause mortality (23) and progression of diabetic nephropathy (14), two outcomes of particular concern in women of reproductive age. Women with T1DM already face substantially elevated lifetime cardiovascular disease risk compared to their non-diabetic peers; pregnancy-associated telomere attrition may compound this risk through effects on vascular endothelial function, accelerated atherosclerotic plaque development, and impaired vascular repair capacity. Telomere shortening in circulating leukocytes has been associated with arterial stiffness, carotid intima-media thickness, and incident coronary events in diabetic populations, suggesting our observations may have prognostic relevance. Our findings, therefore, identify the postpartum period as a potential intervention window where targeted strategies—including optimized glycemic control with minimization of glucose variability, structured weight management programs, evidence-based stress reduction interventions, and screening and treatment for postpartum depression—might mitigate long-term cardiometabolic consequences in this high-risk population. Prospective studies linking postpartum telomere dynamics to subsequent cardiovascular events, renal function decline, and mortality in women with T1DM are warranted to validate the clinical significance of these biomarker changes.

We examined whether glycemic control and maternal BMI influenced telomere dynamics. Our results showed no significant differences in LTL between mothers with HbA1c levels below or above 6% during pregnancy. This finding is consistent with some studies reporting only weak associations between HbA1c and telomere length in T1DM (13), though other work suggests poor glycemic control may contribute to telomere shortening through oxidative stress and inflammation (17, 20). The absence of a significant effect in our cohort may reflect the relatively well-controlled HbA1c values during pregnancy and the limited sample size.

Similarly, maternal BMI showed a non-significant trend toward longer telomeres in normal-weight compared to overweight or obese mothers. Obesity is strongly linked to increased oxidative stress, systemic inflammation, and insulin resistance, all of which are established contributors to telomere shortening (4, 5, 19). While meta-analyses have shown an inverse association between BMI and telomere length in general populations (19, 20), our data suggest that in women with T1DM, the pronounced metabolic and hormonal stressors of pregnancy may overshadow the independent effects of BMI. Larger cohorts are needed to clarify whether maternal weight status meaningfully modulates postpartum telomere dynamics in this high-risk group.

The absence of a significant effect of preterm versus term delivery on maternal LTL at delivery was somewhat unexpected, given that preterm birth is often associated with increased oxidative stress and inflammation. However, the small number of preterm deliveries (n=8) in our cohort likely limited statistical power to detect meaningful differences.

Several limitations of this study warrant consideration. First, the retention rate of 62.2% at one-year follow up raises potential concerns about selection bias, though baseline characteristic did not differ significantly between followed and lost participants (**Table 3**). Second, we did not include a healthy control group, limiting our ability to determine whether the observed telomere shortening exceeds that expected from pregnancy and postpartum alone, contextualization against published literature suggests our finding exceed typical postpartum telomere changes, but direct comparison by methodological heterogeneity. Third, and perhaps most importantly, we lacked systematic data on key confounders including stress, breastfeeding, postpartum weight retention, sleep quality, physical activity, postpartum depression, and postpartum glycemic control, all of which may influence telomere dynamics independently of T1DM status. While quantitative bias analysis suggests unmeasured confounding is unlikely to fully explain our findings, we cannot exclude that lifestyle and psychosocial factors contribute substantially to the observed effect. Fourth, all participants delivered by cesarean section, limiting generalizability to vaginal deliveries. Fifth, the study period partially overlapped with the COVID-19 pandemic, and although direct infections were rare, pandemic-related stress may have influenced telomere dynamics. Finally, our sample size limited statistical power to detect modest effect of clinical variable on telomere shortening.

**Table 3:** Baseline characteristics: followed vs. lost to follow-up.

Variable	Followed (n=46)	Lost (n=28)	P-value
Maternal age (years)	31 (27–34)	30 (25–35)	0.62
T1DM duration (years)	15 (7–21)	14 (5–20)	0.54
Age at T1DM onset (years)	14 (9–23)	15 (10–25)	0.71
Pre-pregnancy BMI (kg/m²)	23.1 (20.5–26.2)	22.6 (20.1–25.8)	0.58
BMI at delivery (kg/m²)	27.8 (25.2–31.3)	27.1 (24.6–30.8)	0.49
Gestational weight gain (kg)	13 (10–16)	12 (9–15)	0.38
HbA1c 1st trimester (%)	6.5 (6.0–7.2)	6.7 (6.1–7.4)	0.44
HbA1c 3rd trimester (%)	6.0 (5.5–6.4)	6.1 (5.6–6.6)	0.52
LTL at delivery (T/S)	2.83 (1.87-4.92)	2.46 (1.74-4.00)	0.31
Gestational age (weeks)	38 (38–38)	38 (37–38)	0.67
Preterm delivery, n (%)	5 (10.9%)	3 (10.7%)	0.98
Neonatal birth weight (g)	3620 (3210–4060)	3580 (3180–4000)	0.73
Graduate education, n (%)	26 (56.5%)	14 (50.0%)	0.59

Data presented as median (IQR) or n (%). P-values from Mann-Whitney U test (continuous) or Chi-square test (categorical).

The strengths of this study include its prospective design, the use of a validated qPCR method for telomere length measurement, and the focus on a well-characterized cohort of women with T1DM receiving standardized prenatal care. The longitudinal measurement of LTL at delivery and one year postpartum provides unique insights into the temporal dynamics of biological aging during this critical life transition.

## 5 Conclusions

Women with T1DM experience significant leukocyte telomere shortening within the first year postpartum. Although maternal HbA1c and BMI did not significantly influence telomere length in this cohort, both remain biologically plausible modifiers of telomere dynamics and warrant further investigation.

These findings highlight the need for personalized postpartum care in women with T1DM. Future multi-center studies including healthy controls, oxidative stress biomarkers, and comprehensive lifestyle assessment should clarify the roles of glycemic control and weight in telomere biology. Interventional approaches aimed at improving metabolic control, weight management, and stress reduction may help mitigate accelerated biological aging in this high-risk population.

## Data Availability

The datasets presented in this study can be found in online repositories. The names of the repository/repositories and accession number(s) can be found below: https://figshare.com/, https://figshare.com/authors/Josip_Djelmis/11937350.
